# Association of COVID-19 Lockdown With Gestational Diabetes Mellitus

**DOI:** 10.3389/fendo.2022.824245

**Published:** 2022-03-30

**Authors:** Zhongrong He, Yanyun Lv, Suijin Zheng, Yudong Pu, Qingmei Lin, He Zhou, Moran Dong, Jiaqi Wang, Jingjie Fan, Yufeng Ye, Hanwei Chen, Rui Qian, Juan Jin, Yumeng Chen, Guimin Chen, Guanhao He, Shouzhen Cheng, Jianxiong Hu, Jianpeng Xiao, Wenjun Ma, Xi Su, Tao Liu

**Affiliations:** ^1^ Guangdong Provincial Institute of Public Health, Guangdong Provincial Center for Disease Control and Prevention, Guangzhou, China; ^2^ School of Public Health, Sun Yat-sen University, Guangzhou, China; ^3^ Affiliated Jiangmen Hospital of Sun Yat-sen University, Jiangmen, China; ^4^ The Affiliated Houjie Hospital, Guangdong Medical University, Dongguan, China; ^5^ Central Laboratory, Songshan Lake Central Hospital of Dongguan City, Dongguan, China; ^6^ Foshan Women and Children Hospital Affiliated to Southern Medical University, Foshan, China; ^7^ School of Public Health, Guangdong Pharmaceutical University, Guangzhou, China; ^8^ Department of Prevention and Health Care, Shenzhen Maternity and Child Healthcare Hospital, Southern Medical University, Shenzhen, China; ^9^ Radiological Department, Guangzhou Panyu Central Hospital, Guangzhou, China; ^10^ Technology Department, Statistical Information Center for Health and Family Planning Bureau of Foshan, Foshan, China; ^11^ School of Public Health, Southern Medical University, Guangzhou, China; ^12^ Nursing Department, The First Affiliated Hospital, Sun Yat-sen University, Guangzhou, China; ^13^ Department of Public Health and Preventive Medicine, School of Medicine, Jinan University, Guangzhou, China; ^14^ Disease Control and Prevention Institute of Jinan University, Jinan University, Guangzhou, China

**Keywords:** COVID-19, lockdown, pregnant woman, gestational diabetes mellitus, China

## Abstract

**Importance:**

The ongoing pandemic of COVID-19 is still affecting our life, but the effects of lockdown measures on gestational diabetes mellitus (GDM) in pregnant women remain unclear.

**Aim:**

To investigate the association between COVID-19 lockdown and GDM.

**Subjects and Methods:**

Medical records of 140844 pregnant women during 2015-2020 were extracted from 5 hospitals in Guangdong Province, China. Pregnant women who underwent the COVID-19 Level I lockdown (1/23 - 2/24/2020) during pregnancy were defined as the exposed group (N=20472) and pregnant women who underwent the same calendar months during 2015-2019 (1/23 - 2/24) were defined as the unexposed group (N=120372). Subgroup analyses were used to explore the potential susceptible exposure window of COVID-19 lockdown on GDM. Cumulative exposure is quantitatively estimated by assigning different weights to response periods with different exposure intensities. A logistic regression model was used to estimate the association between COVID-19 lockdown exposure and GDM.

**Results:**

The rates of GDM in the exposed and unexposed groups were 15.2% and 12.4%, respectively. The overall analyses showed positive associations (odds ratio, OR=1.22, 95%CI: 1.17, 1.27) between lockdown exposure and GDM risk in all pregnant women. More pronounced associations were found in women who underwent the COVID-19 lockdown in their first four months of pregnancy, and the adjusted OR values ranged from 1.24 (95%CI: 1.10, 1.39) in women with 5-8 gestational weeks (GWs) to 1.35 (95%CI: 1.20, 1.52) with < 5 GWs. In addition, we found a positive exposure-response association of cumulative lockdown exposure with the risk of GDM.

**Conclusions:**

The COVID-19 lockdown was associated with an increased risk of GDM, and the first four months of pregnancy may be the window for sensitive exposure.

## Introduction

Gestational diabetes mellitus (GDM) is temporary hyperglycemia induced by glucose intolerance with onset or first monitor during pregnancy (1). As one of the most common complications in pregnant women, GDM is widely prevalent around the world. The median estimated prevalence of GDM in the Middle East and North Africa region is 12.9% versus 5.8% in Europe, while in the Western Pacific region, prevalence estimates vary from 4.5% in Japan to 25.1% in Singapore (2). In China, the prevalence of GDM is also not optimistic. A meta-analyses conducted by Gao et al. in 2019 found a prevalence of GDM of 14.8% across China (3). According to the data released by the International Diabetes Federation (IDF), more than 1 million Chinese women were affected by GDM in 2013, ranking second in the world after India (4).

Although the degree of blood glucose elevation is usually not as high as that of diabetes mellitus combined with pregnancy, it can still cause serious harm to both women and fetuses (4). The short-term effects of GDM on mothers and infants include increased maternal pregnancy complications, such as gestational hypertensive disease and polyhydramnios, as well as increased risk of fetal macrosomia, and neonatal respiratory distress syndrome (4). The long-term threat to maternal and child health is mainly the increased risk of long-term type 2 diabetes mellitus (T2DM) and metabolic syndrome in mothers after postpartum and offspring (2, 4, 5). Thus, reducing the prevalence of GDM is an important public health issue.

Since the early 2020, the COVID-19 pandemic and the corresponding catastrophic effect have challenged the view of public health of the world’s people. At the time of the COVID-19 pandemic, a series of special measures have been adopted by governments and health-care leaders around the world to decrease the pandemic of the virus. For example, many cities and regions have fully or partially implemented lockdown measures, with large venues closed and traffic restricted. Apart from the control of pandemic, these measures during the lockdown have not only led to economic recessions, but also the strain on medical resources (6–8), which has substantially affected health in the public such as glycemic control in diabetic patients (9, 10).

Pregnant women go through huge physiological and psychological changes during pregnancy, and are more potentially affected by extreme events (11). A few epidemiological studies have reported significant associations of COVID-19 lockdown with maternal health and pregnancy outcomes, such as stillbirth, and preterm delivery (12, 13). While as a common complication of pregnancy, few studies have assessed the association of COVID-19 lockdown measure with GDM. A study had found the association between COVID-19 and blood sugar control in pregnant women (14). Moreover, there are several research issues or limitations that need to be fully addressed or investigated in future studies. First, previous studies focused on changes in glycemic control in patients with GDM and changes in GDM prevalence during COVID-19 need to be further evaluated. Second, the impact of environmental changes on maternal health is related to the stage of pregnancy (15, 16). The sensitive window exposure period when COVID-19 affects pregnant women’s GDM remains unknown. Third, the intensity and duration of the lockdown varied constantly, and its impact on GDM should be considered.

Accordingly, to fill in these research gaps, we comprehensively evaluated the association between the COVID-19 lockdown and the risk of GDM by quantifying the duration and intensity of exposure among pregnant women in Guangdong Province, South China. Moreover, we considered seasonal effects and adequate follow-up time in this study.

## Materials and Methods

### Subjects

**Figure 1:**
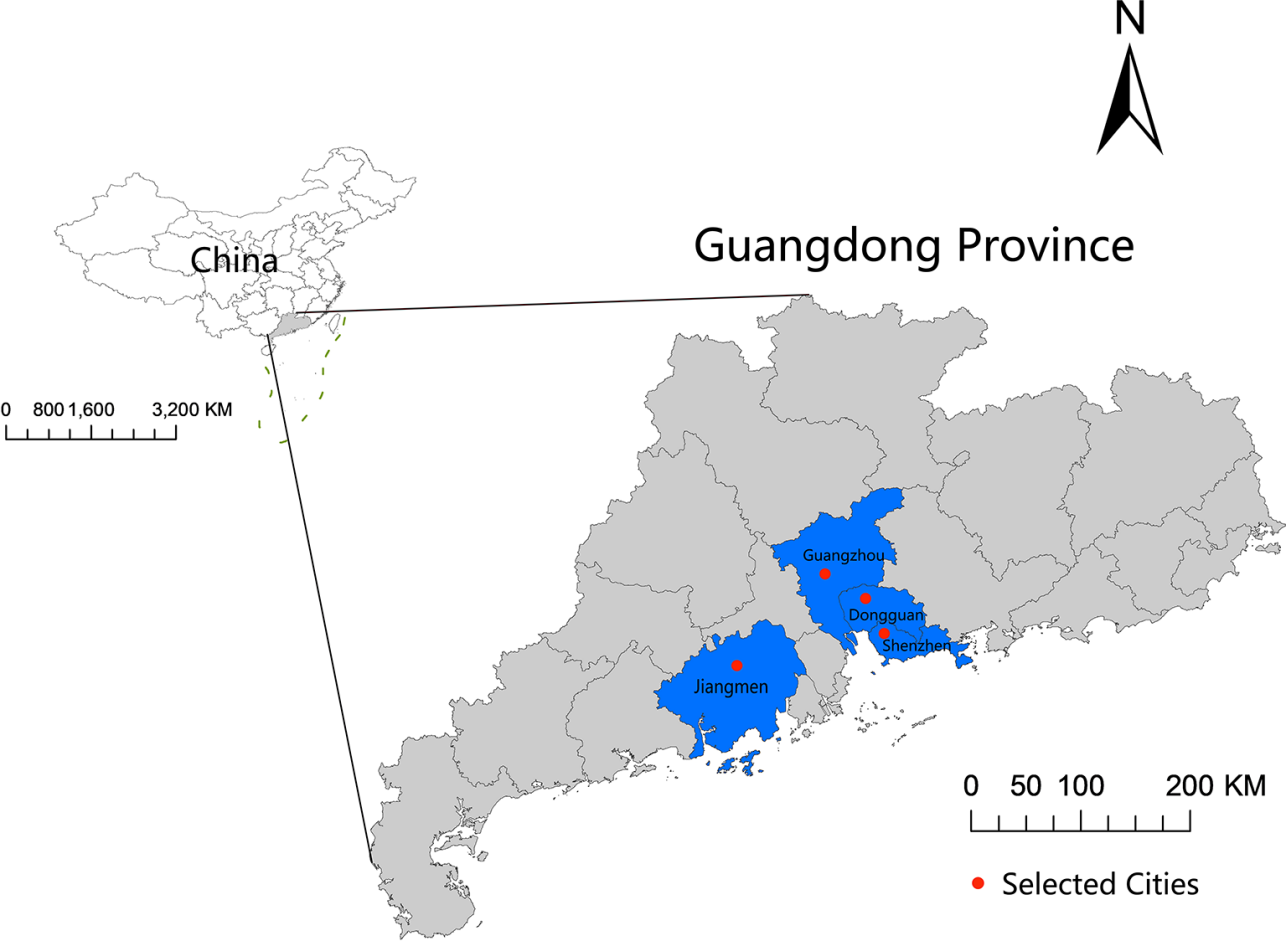
Geographic locations of the four included study cities in Guangdong Province, China.

Medical records of 222126 pregnant women between 2015 and 2020 were obtained from 5 hospitals [Guangzhou (n=1), Shenzhen (n=1), Dongguan (n=2), and Jiangmen (n=1)] located in Guangdong Province, China (**Figure 1**). We excluded 1318 women with missing information on important variables, and 37487 women whose gestational period did not overlap with the COVID-19 lockdown period in 2020 or the same period in 2015-2019. Because GDM is usually diagnosed during the 24th to 28th gestational week (GW) (17), we further excluded those (n=42353) pregnant women whose gestational age was larger than 28GWs at the time of COVID-19 lockdown and the corresponding period in 2015-2019, and those (n=124) preterm pregnant women with less than 28 GWs (not screened for GDM) when had their childbirth. Finally, 140844 pregnant women were included in the data analyses. None of these pregnant women was infected with SARS-CoV-2 (**Supplementary Figure 1**).

### Data Collection

We extracted the following individual information from maternal medical records: maternal age, gestational weeks (GWs), marital status, parity, and gestational diabetes mellitus (GDM). For this study, the data is imported into R3.6.1 software to clean up the data information mentioned above. Unreasonable or abnormal values were either amended or defined as missing.

### Exposure Assessment

According to the National Emergency Plan for Public Emergencies, the emergency response for public health emergencies is divided into four levels: Level I (especially serious), Level II (serious), Level III (relatively serious), and Level IV (general) (18). In response to the COVID-19 outbreak, Guangdong Province launched a Level I response on 1/23/2020. Then the public health emergency response level was adjusted to Level II on 2/24/2020 and Level III on 5/9/2020. During the Level I response, the government implemented control measures to minimize public gatherings and stopped public gatherings rigorously (19). Shopping malls, bars, schools, and other establishments were closed, and traffic was restricted. After the Level I response, fewer restrictions were imposed. During the Level II response, public places at risk of cross-infection were temporarily closed or disinfected. During the Level III response, except for masks and temperature checks in certain places such as hospitals and shopping malls, life is gradually returning to pre-pandemic conditions (**Supplementary Table 1**).

The days with Level I response (1/23-2/24/2020) were defined as Level I lockdown. We identified the exposure group (N=20472) based on the time of pregnancy crossed with the Level I lockdown. The unexposed group (N=120372) experienced the same calendar months as the exposed group between 2015 and 2019. This helps to control for seasonal effects, as our data suggest that the GDM rate is related to the season of pregnancy (**Supplementary Figure 2**).

To explore the potential susceptible exposure window, we divided the exposed group into 8 subgroups according to GWs and the crossover of 1/23/2020. We calculated the date of conception based on GWs and birth date. For instance, the first subgroup consisted of women who conceived during the Level I lockdown, and the second group was pregnant women in the first four GWs of gestational age at 2020/1/23 (**Supplementary Figure 3**). The grouping ended at 28 GWs. Similarly, the unexposed group was also divided into 8 subgroups (considering the same calendar month) and matched with the exposed group. For each pair of subsets (exposed vs unexposed), we calculated the associations between lockdown exposure and GDM risk.

**Figure 2:**
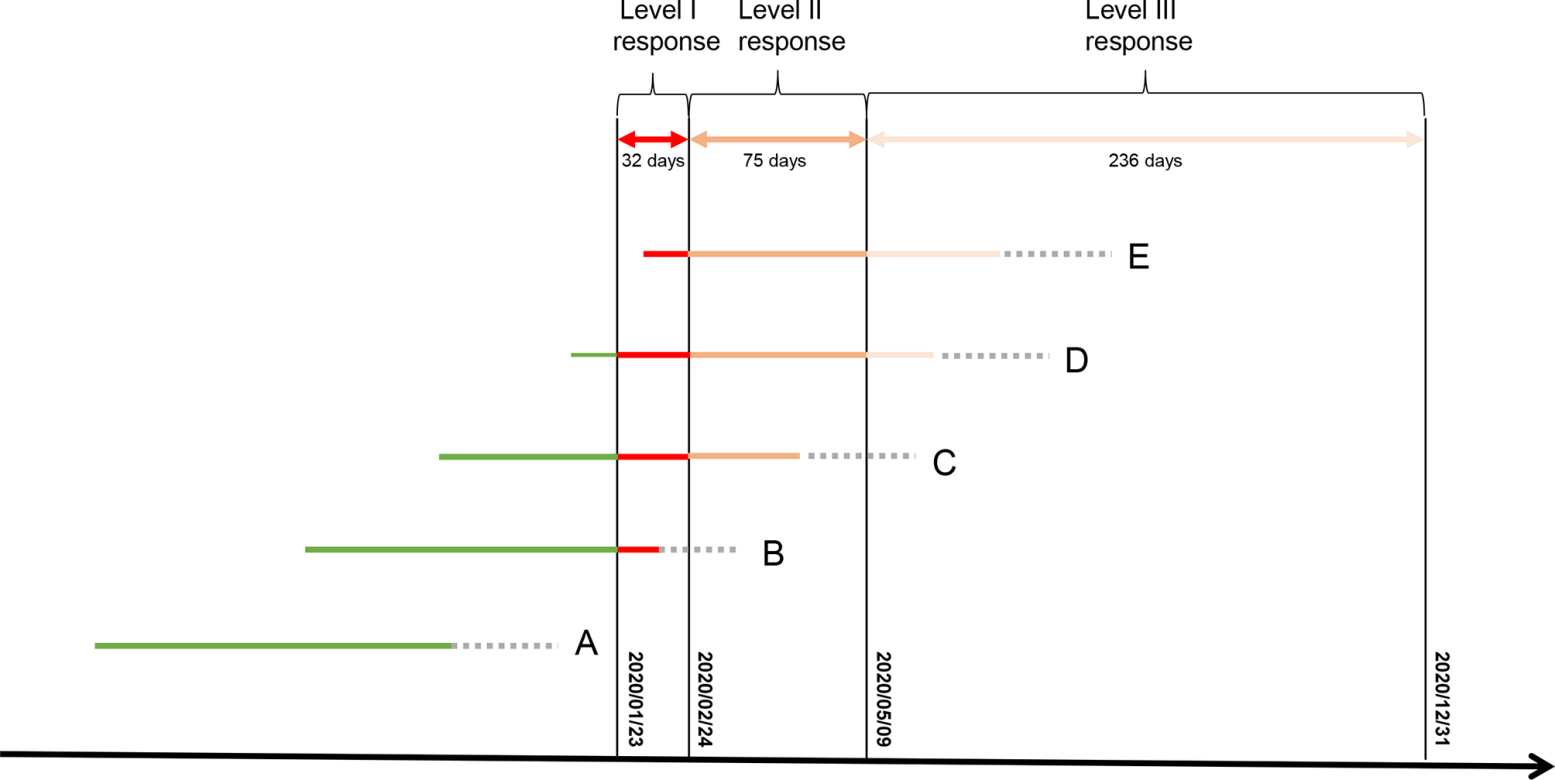
Approach to calculating individual cumulative exposure dose to lockdown in the first 28 GWs. Weeks after 28 GWs. **(A–E)** represent subgroups of pregnant women with different GWs during the Level I lockdown; We assigned a weighting value of 3 to the days with Level I response, 2 to the days with Level II response, 1 to the days with Level III response, and 0 to days before lockdown (no exposure).

The lockdown measures during the Level II (2/25-5/9/2020) and Level III (5/10-12/31/2020) responses might also adversely affect GDM risk. Therefore, we assigned different weights to different response times, and multiply response times with weights to quantitatively estimate the cumulative exposure: (no response, weighting=0), (Level I, weighting=3), (Level II, weighting=2), and (Level III, weighting=1). Because GDM is usually diagnosed between 24 and 28 GWs (17), we only estimated the amount of cumulative exposure before 28 GWs (**Figure 2**). **Supplementary Figure 4** shows the distribution of cumulative exposures to COVID-19 lockdown.

### Outcome Measures

Individual information on GDM was extracted from each woman’s medical record. Gestational diabetes was diagnosed when the blood glucose was higher than the standard at any of the three time points: fasting blood glucose 5.1 mmol/L; 1-h plasma glucose 10.0 mmol/L following a 75 g oral glucose tolerance test; or 2-h plasma glucose 8.5 mmol/L following a 75 g oral glucose tolerance test (OGTT) (20).

### Potential Confounders

We probe into the following confounders potentially associated with GDM based on biological plausibility, literature review, and data availability: maternal age, marital status, parity, and residential city.

### Statistical Analyses

Chi-square test (for categorical variables) or *t-*test (for continuous variables) were applied to detect the difference in the distribution of maternal characteristics between exposed (n = 20472) and unexposed (n = 120372) groups. An unconditional logistic regression model was implemented to estimate the associations of lockdown exposure with GDM, after adjusting for potential confounders. The logistic regression model was also implemented to analyze the association between the cumulative exposure dose and GDM risk. The cumulative exposure was treated as a continuous variable and categorical variables in the logistical regression model. For the categorical variable, the cumulative exposure was divided into four groups [Q1 (<25% centile), Q2 (≥25% centile and <50% centile), Q3(≥50% centile and <75% centile), and Q4 (≥75% centile)] according to the quartiles. The trend test is performed by inputting the four groups as continuous variables. We employed a generalized additive model (GAM) with a binomial link function to estimate the potential nonlinear exposure-response association between cumulative lockdown exposure and the risk of GDM. A penalized smoothing spline with 3 degrees of freedom (df) was used to estimate the potential nonlinear effect of cumulative lockdown exposure.

### Sensitivity Analysis

It was reported that the worldwide prevalence of GDM is constantly increasing (3). Thus, it is expected that prevalence of 2020 may be significantly higher of those of 2015, independently from lockdown. To test the potential impact of long-term trend of GDM prevalence on the association between COVID-19 lockdown and GDM risk, we selected those pregnant women only in 2019 as the control group.

'We performed all the analyses in R3.6.1 (R Development Core Team 2019). And all p values were 2-sided, and a *P*-value <0.05 was considered statistically significant.

### Ethics Statement

This study was approved by the Ethics Committee of Guangdong Provincial Center for Disease Control and Prevention (No. W96-027E-2020004). Written informed consent was obtained from all participants.

## Results

### General Characteristics of Study Subjects

Out of the total included 140844 pregnant women, 20472 were identified as exposed group and 120372 were defined as unexposed group (**Table 1**). Compared with the unexposed group, the exposed group had a significantly higher proportion of women aged 30 years or older (59.5% vs 54.1%) and a lower proportion of married (94.5% vs 96.6%).

**Table 1:** General characteristics of study participants.

	Unexposed group (n = 120372) No. of participants (%)	Exposed group (n = 20472) No. of participants0 (%)	*χ* ^2^	*P*
**Maternal age (years)**				
<24	8670 (7.2)	1150 (5.6)	228.61	<0.001
24–26	17309 (14.4)	2596 (12.7)		
27–29	29234 (24.3)	4546 (22.2)		
30–32	26770 (22.2)	5089 (24.9)		
33–35	20511 (17.0)	3696 (18.0)		
>35	17878 (14.9)	3395 (16.6)		
**Residential city**				
Guangzhou	15381 (12.8)	2346 (11.5)	37.267	<0.001
Dongguan	27843 (23.1)	4624 (22.6)		
Jiangmen	15230 (12.7)	2725 (13.3)		
Shenzhen	61918 (51.4)	10777 (52.6)		
**Gestational diabetes mellitus (GDM)**			123.449	<0.001
No	105413 (87.6)	17352 (84.8)		
Yes	14959 (12.4)	3120 (15.2)		
**Marital status**			750.81	<0.001
Married	116223 (96.6)	19346 (94.5)		
Unmarried	3403 (2.8)	598 (2.9)		
Other	746 (0.6)	528 (2.6)		
**Parity**			2.6951	0.260
0 (Primiparas)	57293 (47.6)	9858 (48.2)		
1 (Multiparas)	50676 (42.1)	8559 (41.8)		
2-4 (Multiparas)	12403 (10.3)	2055 (10.0)		
	**Mean ± SD**	**Mean ± SD**	***t***	***P***
**Maternal age (years)**	30.31 ± 4.85	30.80 ± 4.84	13.462	<0.001

### Associations of COVID-19 Lockdown Exposure With GDM

We observed a greater prevalent GDM in the exposed group (15.2%) than the unexposed group (12.4%). Multivariable analyses showed a positive association [adjusted odds ratio (OR)= 1.22, 95%CI: 1.17, 1.27] of lockdown exposure with GDM in the total pregnant women, after adjustment for maternal age, marital status, parity, and residential city. Subgroup analyses showed that the significant associations were only found in pregnant women who experienced the Level I lockdown in the first four months of pregnancy. The adjusted ORs varied from 1.35 (95%CI: 1.20, 1.52) in women with less than 5 GWs to 1.24 (95%CI: 1.10, 1.39) in women with 5-8 GWs on 1/23/2020, the beginning of Level I response (**Table 2**).

**Table 2:** Associations of exposure to the COVID-19 lockdown with gestational diabetes mellitus.

	Unexposed group (n, %)		Exposed group (n, %)^a^		OR for GDM (95%CI)	
	GDM (-)	GDM (+)	GDM (-)	GDM (+)	Crude OR(95% CI)	Adjusted OR^*^ (95% CI)
Gestational week at the beginning of the Level I lockdown						
All	105413 (87.6)	14959 (12.4)	17352 (84.8)	3120 (15.2)	1.27 (1.22, 1.32)	1.22 (1.17, 1.27)
Conception during the lockdown	16228 (87.6)	2298 (12.4)	2271 (84.0)	432 (16.0)	1.34 (1.20, 1.50)	1.30 (1.16, 1.46)
Prior to 5th	13431 (87.6)	1905 (12.4)	2229 (83.5)	439 (16.5)	1.38 (1.24, 1.55)	1.35 (1.20, 1.52)
5th -8th	13188 (86.9)	1988 (13.1)	2293 (83.9)	441 (16.1)	1.27 (1.14, 1.43)	1.24 (1.10, 1.39)
9th -12nd	12881 (87.8)	1783 (12.2)	2159 (84.8)	387 (15.2)	1.29 (1.15, 1.46)	1.25 (1.11, 1.41)
13rd -16th	12743 (88.6)	1643 (11.4)	2332 (86.0)	379 (14.0)	1.26 (1.12, 1.42)	1.26 (1.11, 1.42)
17th -20th	12946 (87.9)	1787 (12.1)	2187 (86.6)	337 (13.4)	1.11 (0.98, 1.26)	1.04 (0.92,1.19)
21st -24th	12058 (87.3)	1760 (12.7)	2036 (85.3)	352 (14.7)	1.18 (1.05, 1.34)	1.11 (0.97, 1.26)
25th -28th	11938 (86.9)	1795 (13.1)	1845 (83.9)	353 (16.1)	1.27 (1.12, 1.44)	1.20 (1.06, 1.36)

*Adjusted for maternal age, marital status, parity, residential city.

GDM, gestational diabetes mellitus.

a Pregnant women who have experienced the COVID-19 lockdown (from 1/23/2020 to 2/24/2020) during any period of their pregnancy were defined as the exposed group. We further divided the exposed group into subgroups according to their gestational weeks (GW) on 1/23/2020, the beginning of lockdown.

### Association of Cumulative Exposures to COVID-19 Lockdown With GDM

**Figure 3:**
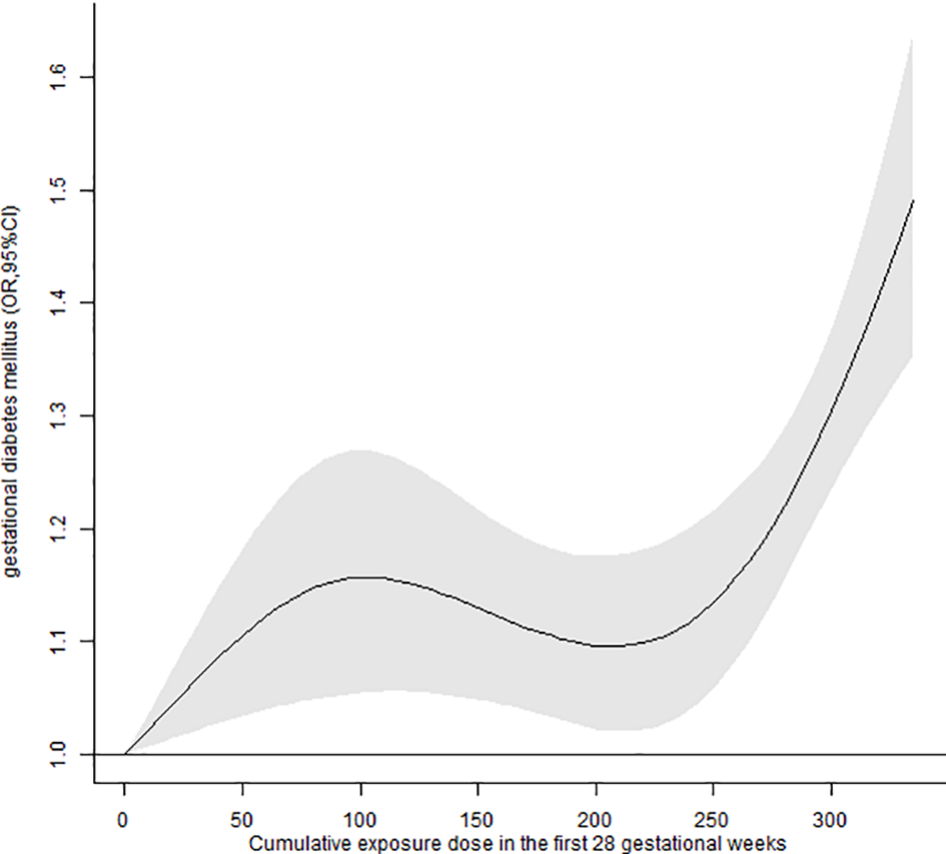
Associations between cumulative exposure dose to lockdown in the first 28 GWs and the risk of GDM.

We also found significant positive associations between cumulative exposure dose and GDM risk (**Table 3**). The risk of GDM increased by 1.09 (95%CI:1.07, 1.11) times for each additional 100 units of cumulative exposure during the first 28 GWs. Compared with the unexposed group, the adjusted ORs of GDM in the Q1, Q2, Q3, and Q4 groups were 1.17 (95%CI: 1.08, 1.27), 1.10 (95%CI: 1.02, 1.20), 1.22 (95%CI: 1.13, 1.32), and 1.39 (95%CI: 1.29, 1.50), respectively. In addition, the nonlinear exposure-response relationship showed that higher cumulative lockdown exposure was associated with a higher risk of GDM (**Figure 3**).

**Table 3:** Associations of cumulative exposure to the COVID-19 lockdown with gestational diabetes mellitus.

	Exposure dose in	Exposure dose in				OR for GDM (95%CI)	
	Unexposed group (Mean ± SD)	Exposed Group (Mean ± SD)		No. of participants (%)		Crude OR (95% CI)	Adjusted OR^*^ (95% CI)
	GDM (-) +GDM (+)	GDM (-)	GDM (+)	GDM (-)	GDM (+)		
**Cumulative exposure dose in the first 28 weeks during the Level I to the Level III lockdown** **^a^**							
Per 100 unit increase in all participants	0 ± 0	223.94 ± 90.67	227.11 ± 92.55			1.10 (1.08, 1.12)	1.09 (1.07, 1.11)
**Categories of cumulative exposure dose**							
Unexposed group	0 ± 0	–	–	105413 (87.6)	14959 (12.4)	Reference	Reference
Q_1_ (<158)	–	89.35 ± 44.45	86.75 ± 43.89	4310 (84.9)	769 (15.1)	1.26 (1.16, 1.36)	1.17 (1.08, 1.27)
Q_2_ (158-256)	–	211.19 ± 29.87	213.54 ± 30.62	4448 (86.1)	720 (13.9)	1.14 (1.05, 1.24)	1.10 (1.02, 1.20)
Q_3_ (257-298)	–	279.07 ± 11.64	279.16 ± 11.48	4306 (84.9)	763 (15.1)	1.25 (1.15, 1.35)	1.22 (1.13, 1.32)
Q_4_ (≥299)	–	317.10 ± 10.74	316.98 ± 10.97	4288 (83.2)	868 (16.8)	1.43 (1.32, 1.54)	1.39 (1.29, 1.50)
* P* for trend test							< 0.001

*Adjusted for maternal age, marital status, parity, residential city.

GDM, gestational diabetes mellitus.

a The exposed group refers to the pregnant women who have experienced the COVID-19 lockdown in their first 28 GWs. The other participants were defined as the unexposed group. The individual cumulative exposure dose was calculated by combining the weightings with the overlap between their pregnancy period ≤28 GWs and the three levels of responses. Q_1_-Q_4_ were defined as the cumulative exposure dose of the exposed group classified by quartiles, and the unexposed group was used as reference.

-Not applicable.

### Sensitivity Analyses

The results of sensitivity analysis suggest that the associations between COVID-19 lockdown and GDM were attenuated, and subgroup analyses suggested that the significant association was found only during the first five GWs (**Supplementary Table 2**). However, the adjusted OR of GDM in all pregnant women was also statistically significant (1.07, 95%CI: 1.02, 1.14), which indicated the solid effect of COVID-19 lockdown on the risk of GDM.

## Discussion

This study comprehensively investigates the effect of COVID-19 lockdown measures on GDM risks in pregnant women using a large database from South China. The results suggested that the COVID-19 lockdown measures were associated with an increased risk of GDM in pregnant women. The association was stronger in pregnant women within the first four months of pregnancy during the Level I lockdown period. In addition, we observed a significant exposure-response association between cumulative exposures to lockdown and GDM risk. These findings extend our understanding of the effects of COVID-19 lockdown measures on maternal and fetal health, and suggest taking actions to prevent the risk of GDM in pregnant women during COVID-19 lockdown periods.

A population study in Italy is consistent with our results. Zanardo et al. found a significant increase in the prevalence of GDM among pregnant women during the COVID-19 pandemic. Experiencing lockdown during the first trimester of pregnancy plays an important role in increasing the GDM risk in pregnant women (21). Moreover, several previous studies had estimated the associations of disasters or the COVID-19 pandemic with adverse human health including pregnancy complications. For example, a study in New York State reported an increased risk of GDM after massive power outages during Hurricane Sandy (22). Another study found a 42.3% (95% CI: 15.0%, 76.0%) increase in emergency department visits for diabetes or abnormal blood sugar in New York State during Hurricane Sandy (23). A study of the Great East Japan Earthquake of 2011 showed a 5% increase in the prevalence of GDM among the most affected residents compared to those who were not affected (24). On top of that, during the COVID-19 lockdown, an Indian cohort study found an increased risk of type 2 diabetes (25), and some other studies found that lockdown measures designed to avoid SARS-CoV-2 transmission may contribute to the deterioration of control in patients with diabetes (9, 10).

These previous studies suggest the plausible causal association between COVID-19 lockdown and GDM, which may relate to several reasons. First, during the COVID-19 lockdown period, most medical services were allocated to tackle the pandemic, and it is difficult for pregnant women to receive timely and adequate prenatal care (26). Pregnant women may also cut back on prenatal care for reasons such as fear of contracting COVID-19 patients in the hospital, following government recommendations to stay home, and restricting transportation (27, 28). Second, social distancing and family economic stress during the lockdown may induce psychological problems in pregnant women who could not attend entertainment venues, play team sports, or meet friends to relax (7, 29). Mental disorders have been regarded as a common risk factor of GDM (30). Third, there is a lot published data, including from China (31), to show that people gain weight during the lockdown. Maternal BMI was an independent risk factor for GDM (32). During the lockdown, snacks and carbohydrates are consumed more (33, 34), and the movement range and mode were greatly restricted (14, 35), which can lead to an elevated maternal BMI.

We further observed that women in the first four months during the Level I lockdown were at a greater risk of developing GDM, which is consistent with previous studies. For instance, Abdo et al. also reported a positive association between exposure to wildfire smoke during early pregnancy and GDM (36). These findings suggest that early pregnancy might be a susceptible exposure window for environmental factors affecting GDM in pregnant women. Changes in environments, behaviors, and the psychological status during the lockdown, such as physical inactivity, low sleep levels, poor diet, and mental health problems, may disturb the normal glycometabolism, and lead to GDM (37). In addition, these women in the early pregnancy during the Level I lockdown would continue to experience lockdown measures even though the Level I lockdown was over, and therefore get more cumulative exposures to lockdown measures in the first 28 GWs. We also observed a positive exposure-response association between cumulative exposure to COVID-19 lockdown and the risk of GDM, which also suggests a higher risk of GDM in women who have experienced the most cumulative exposures to lockdown. Therefore, the government and others should consider how to provide economic, medical treatment, and psychological assistance to pregnant women to reduce the risk of GDM.

### Strengths and Limitations

There are several strengths in this study. First, this is the first study to quantitatively assess the exposure to COVID-19 lockdown, and investigate the association with GDM risk in a Chinese population of pregnant women. We not only estimated the association of exposure to COVID-19 lockdown as an event with the risk of GDM but also provided the exposure-response association between cumulative exposure to lockdown and GDM risk. Second, we applied a large dataset with detailed individual information to investigate the association between lockdown and GDM risk. The dataset covered a wide enough timespan, in which GDM information of all women who have experienced the lockdown was recorded. The large sample size also provided us an adequate statistical power to implement subgroup analyses and identify the potential susceptible exposure window. Third, we used strict contemporaneous controls to reduce the impact of seasonal effects on the occurrence of GDM. To test the seasonal impacts, we estimated the difference in GDM rates between the exposed group and all pregnant women in 2015-2019 (rather than matching calendar months). After adjustment for maternal age etc., we found no statistical association between lockdown and GDM risk (**Supplementary Table 3**). These strengths could provide a stronger causal argument for our findings.

Several limitations should be considered. First, our study is a retrospective study, due to the unexpected emergence of the COVID-19 and the related lockdown measures, which limited our ability to infer the causal relationship between lockdown and GDM. Second, information of all participants was extracted from their medical recodes. Hence, several individual covariates such as maternal BMI, heredity for T2DM, smoking, alcohol consumption were not obtained in this study, and the influence of these confounding factors on the association was unknown. Third, the COVID-19 lockdown measures were implemented across countries with substantial variation in timing, content, and comprehensiveness. But this study was conducted in only four south cities, which limits the generalization of our findings. Fourth, some countries used alternative criteria for diabetes screening to avoid pregnant women staying in the hospital for long time during the COVID-19 pandemic (38, 39). However, it was not clear whether the diagnostic criteria for GDM were modified during the lockdown in this study, which may be a potential bias in this study. Some studies reported that using alternative criteria can increase the missed diagnosis rate of GDM by as much as 30-50% (40, 41). Meanwhile, a prospective study by Molina-Vega et al. found that the rate of missed diagnosis of GDM did not substantially change when comparing conventional criteria used before the pandemic with alternative diagnostic criteria used during the COVID-19 pandemic (42). Therefore, more studies are needed to examine the effects of diagnosis criteria on the association between COVID-19 lockdown and GDM. Fifth, the COVID-19 lockdown included many measures which were usually implemented simultaneously. As a result, we cannot determine their individual impact on the risk of GDM. The COVID-19 is still ongoing throughout the world, and the lockdown measures have been implemented in many countries. Therefore, more research works are needed to demonstrate the effect of COVID-19 lockdown measures on GDM.

In conclusion, we found that the COVID-19 lockdown was associated with a moderately higher risk of GDM, and the first four months might be a susceptible exposure window. Now that the global pandemic of COVID-19 is not over, and we are also confronted with the challenge of the Delta variant B.1.617.2. A study had shown that non-pharmaceutical interventions have made a huge difference in controlling the epidemic (43), so that the lockdown measures will continue to affect our lives. Our findings suggest the critical importance of planning for strong maternal services in the future lockdown. Governments and women’s health care providers must take action to reduce the risk of pregnant women developing GDM. Given the nature of this study, more investigation is needed to clarify the association between the lockdown measures and GDM, which is critical to maternal health.

## Data Availability Statement

The original contributions presented in the study are included in the article/**Supplementary Material**. Further inquiries can be directed to the corresponding authors.

## Author Contributions

TL and XS conceived study hypotheses. ZH, YL, SZ, YP, and QL conceptualized and designed the study. TL, XS, ZH, and YL edited the first draft of the manuscript. ZH, HZ, MD, JW, JF, YY, and HC did formal analyses, interpreted the results. RQ, JJ, YC, GC, GH, and SC contributed to data curation and did statistical analyses. JH, JX, and WM helped interpret and discuss the results. All authors critically revised and approved the final manuscript.

## Funding

The study was funded by the National Natural Science Foundation of China (81874276, 42175181); Natural Science Foundation of Guangdong Province (2019A1515011264); Key-Area Research and Development Program of Guangdong Province (2019B111103001); Science and Technology Program of Guangzhou (202102080565); Chinese Postdoctoral Science Foundation (2020T130020ZX); and Foshan Key Technology Project for COVID-19 (2020001000376). These funders had no role in the design of the study, data collection, and analyses, or preparation of the manuscript.

## Conflict of Interest

The authors declare that the research was conducted in the absence of any commercial or financial relationships that could be construed as a potential conflict of interest.

## Publisher’s Note

All claims expressed in this article are solely those of the authors and do not necessarily represent those of their affiliated organizations, or those of the publisher, the editors and the reviewers. Any product that may be evaluated in this article, or claim that may be made by its manufacturer, is not guaranteed or endorsed by the publisher.
