# Supplementary material for: Association of COVID-19 Lockdown With Gestational Diabetes Mellitus
Source: Front Endocrinol (Lausanne). 2022 Mar 30;13:824245. doi: 10.3389/fendo.2022.824245 (PMC9005639; doi:10.3389/fendo.2022.824245)
Supplement: Supplementary file 1 [file DataSheet_1.doc]

**Supplemental materials**

**Supplementary tables**

**Supplementary Table 1. The prevention and control measures of three level response in Guangdong province**

| **The levels of emergency response** | **Prevention and control measures** | **Weighting**  **score** |
| --- | --- | --- |
| **Level I response** | - Body temperature monitoring and information registration implemented at the entrance of communities and opened public places like shops. Traffic quarantine inspection strictly implemented at airports, wharves, and inter-provincial bus stations. Cleaning and sanitation of key places such as markets to eliminate the possible breeding places for the virus. - Residents’ social movement and gathering rigorously restricted. Masks mandated to be worn in public transportation like buses, taxis, trains, and all crowded public places. - Strong management of COVID-19 patients, suspected patients, and close contacts. The "four centralized" prevention, control, and treatment measures adopted: centralized patients, centralized experts, centralized resources, and centralized treatment. Symptom surveillance was strictly carried out of people who entered Guangdong Province from other places. Medical supplies and safety equipment ensured, such as ambulances, disinfectants, testing reagents, medical devices, and protective equipment. The production and allocation of urgently needed materials promoted. | 3 |
| **Level II response** | - Some public places (i.e. public transportation, grocery stores, and gas station) and residential communities reopened. Shopping malls, cinemas, internet cafes, and other public places gradually opened up. Body temperature monitoring was continued to be implemented, and people required to maintain social distancing. - Masks required in public transportation (i.e. buses, taxis, trains) and in all crowded public places, but no need to wear a mask in an open area. - Buildings with confirmed or suspected cases closed and quarantined. | 2 |
| **Level III response** | - Social activities, commercial actives, and schools were gradually returned be normal. - All prevention and control measures transferred to new-normal status, including mask-wearing, keeping social distance, and body temperature monitoring in public places. - Symptom surveillance in health care institutes. | 1 |
| **Prior to COVID-19** | - No lockdown measures. | 0 |

**Supplementary Table 2. Associations of exposure to COVID-19 lockdown with GDM risk using a control group of** **pregnant women only in 2019**

|  | **Unexposed group (n, %)** | | **Exposed group (n, %) ^a^** | | **OR for GDM (95%CI)** | |
| --- | --- | --- | --- | --- | --- | --- |
|  | **GDM (-)** | **GDM (+)** | **GDM (-)** | **GDM (+)** | **Crude OR**  **(95% CI)** | **Adjusted OR^*^**  **(95% CI)** |
| Gestational week at the beginning of the Level I lockdown |  |  |  |  |  |  |
| All | 20490 (85.8) | 3403 (14.2) | 17352 (84.8) | 3120 (15.2) | 1.08 (1.03, 1.14) | 1.07 (1.02, 1.14) |
| Conception during the lockdown | 3252 (85.6) | 549 (14.4) | 2271 (84.0) | 432 (16.0) | 1.13 (0.98, 1.29) | 1.12 (0.97, 1.28) |
| Prior to 5th | 2611 (86.4) | 412 (13.6) | 2229 (83.5) | 439 (16.5) | 1.25 (1.08, 1.44) | 1.25 (1.08, 1.45) |
| 5th -8th | 2463 (84.4) | 456 (15.6) | 2293 (83.9) | 441 (16.1) | 1.04 (0.90, 1.20) | 1.05 (0.90, 1.21) |
| 9th -12nd | 2341 (85.9) | 384 (14.1) | 2159 (84.8) | 387 (15.2) | 1.09 (0.94,1.27) | 1.12 (0.96,1.31) |
| 13rd -16th | 2462 (87.09) | 365 (12.9) | 2332 (86.0) | 379 (14.0) | 1.10 (0.94,1.28) | 1.15 (0.98,1.34) |
| 17th -20th | 2517 (85.6) | 425 (14.4) | 2187 (86.6) | 337 (13.4) | 0.91 (0.78,1.06) | 0.87 (0.75,1.02) |
| 21st -24th | 2429 (86.4) | 382 (13.6) | 2036 (85.3) | 352 (14.7) | 1.09 (0.94,1.28) | 1.05 (0.89,1.23) |
| 25th -28th | 2415 (84.9) | 430 (15.1) | 1845 (83.9) | 353 (16.1) | 1.07 (0.92,1.25) | 1.03 (0.89,1.22) |

*: Adjusted for maternal age, marital status, parity, residential city.

GDM: gestational diabetes mellitus

a: Pregnant women who have experienced the COVID-19 lockdown (from 1/23/2020 to 2/24/2020) during any period of their pregnancy were defined as the exposed group and pregnant women who underwent the same calendar months during 2019 (1/23 - 2/24) were defined as the unexposed group. We further divided the exposed group into subgroups according to their gestational weeks (GW) on 1/23/2020, the beginning of lockdown.

**Supplementary Table 3. Associations between exposure to COVID-19 lockdown and GDM risk using a control group of all pregnant women during 2015-2019**

| **Unexposed group (n, %) ^a^** | |  | **Exposed group (n, %) ^b^** | |  | **OR for GDM (95%CI)** | |
| --- | --- | --- | --- | --- | --- | --- | --- |
| GDM (-) | GDM (+) |  | GDM (-) | GDM (+) |  | **Crude OR** | **Adjusted OR ^*^** |
| 166697(87.3) | 24298(12.7) |  | 2212(83.5) | 436(16.5) |  | 1.35(1.22,1.50) | 1.12(0.99,1.26) |

All pregnant women during the level I lockdown period as the exposed group, and all pregnant women during 2015-2019 as the unexposed group.

*: Adjusted for maternal age, marital status, parity, residential city.

GDM: gestational diabetes mellitus

a: All births between 1/1/2015 to 1/22/2020 were defined as the unexposed group.

b: All births during the COVID-19 lockdown (1/23/2020 to 2/24/2020) were defined as the exposed group

**
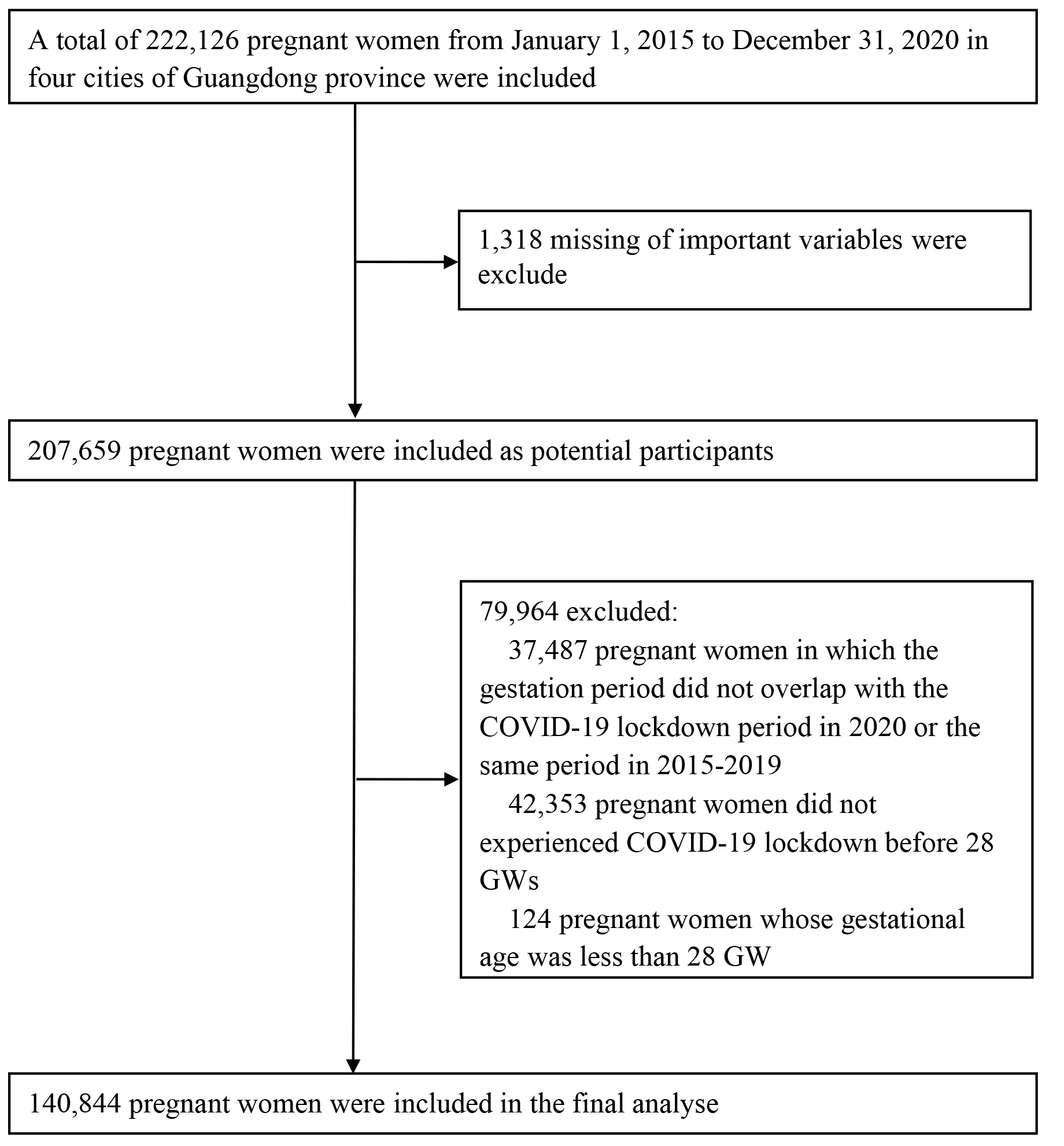
**

**Supplementary Figure 1. Selection process of study subject**


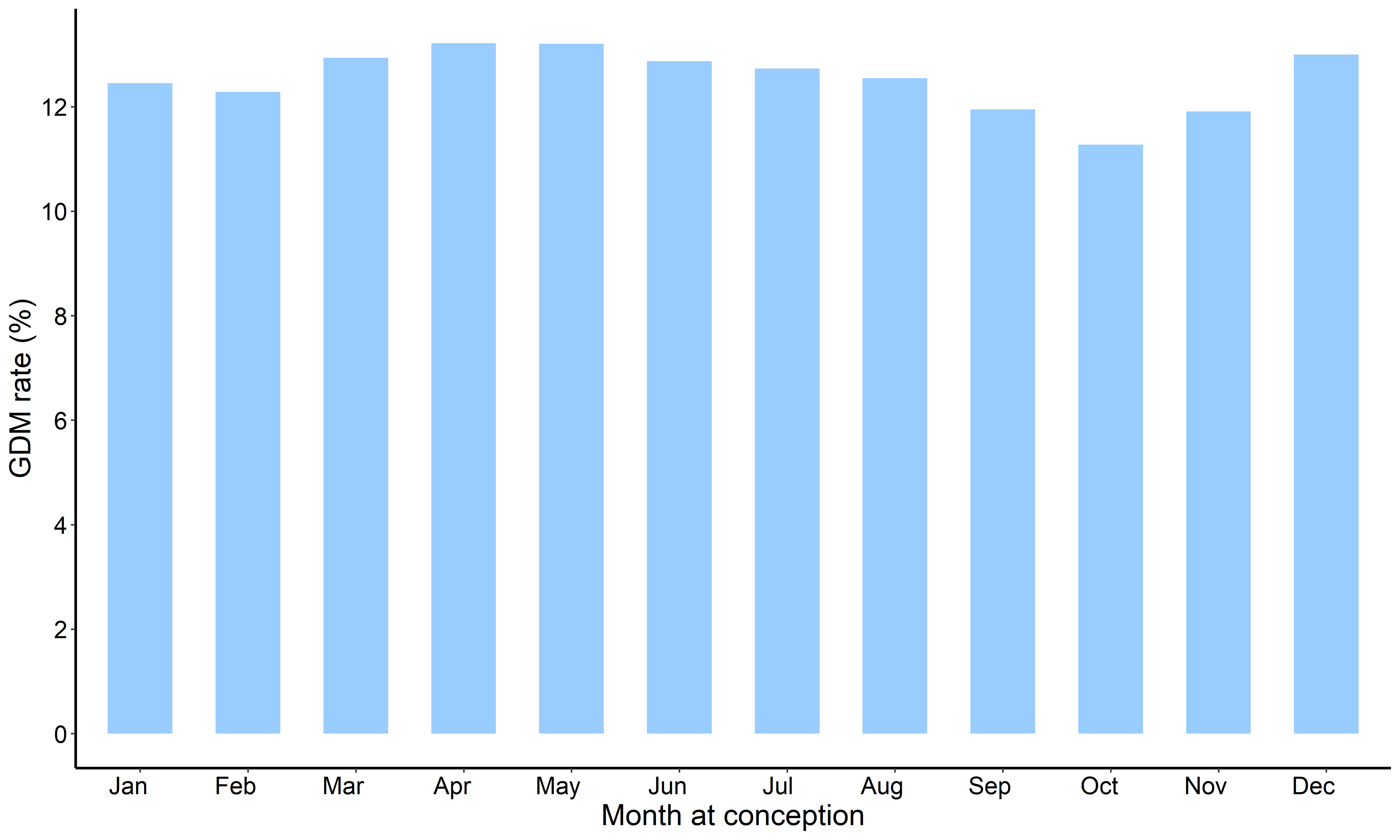


**Supplementary Figure 2. Rate of gestational diabetes mellitus in each calendar month during 2015-2019** **(before the COVID-19 pandemic)**


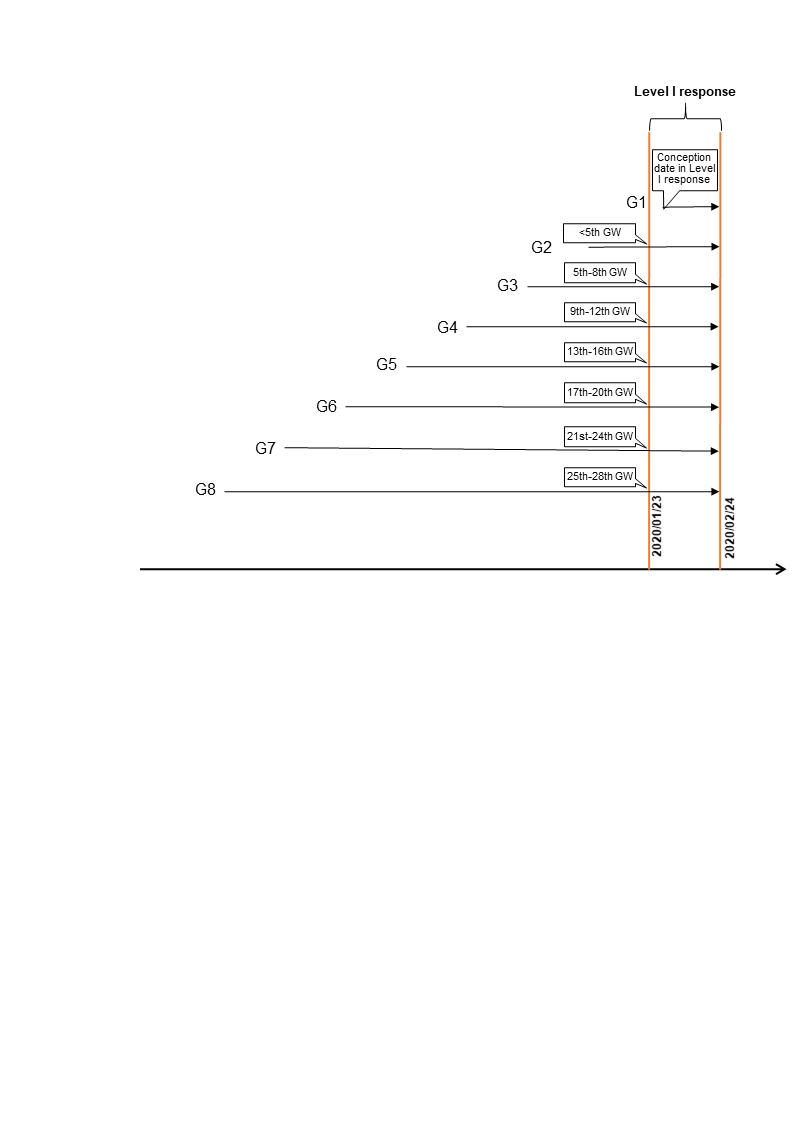


**Supplementary Figure 3. Division of participants into subgroups according to GWs and the crossover of 1/23/2020**

Note: G1-G8: Eight subgroups; 1st-28th GW represent GWs on 1/23/2020.

**
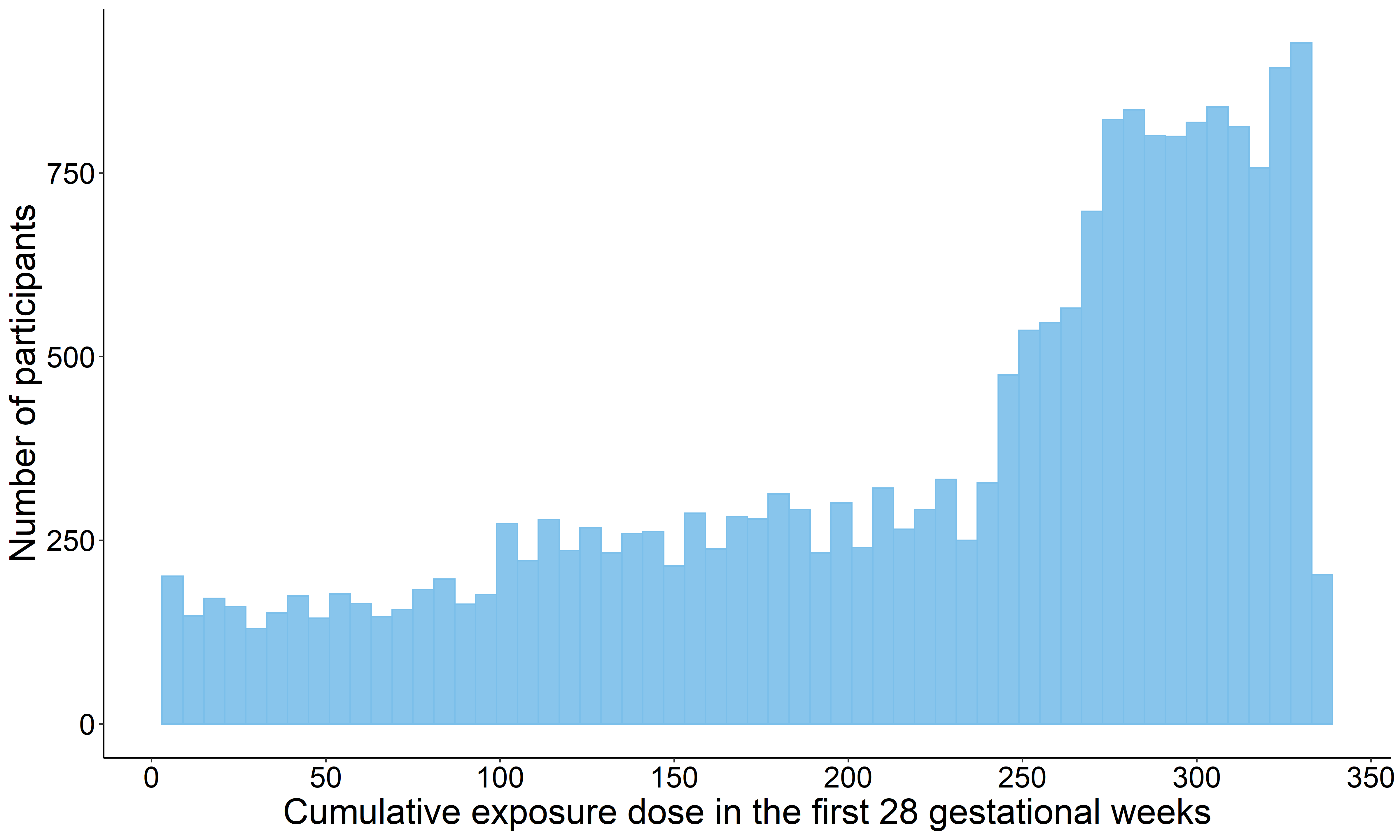
**

**Supplementary Figure 4. Distribution of the cumulative exposure dose in the first 28 GWs in the exposed group during lockdown**
